# The arterial blood pressure associated with terminal cardiovascular collapse in critically ill patients: a retrospective cohort study

**DOI:** 10.1186/s13054-014-0719-2

**Published:** 2014-12-19

**Authors:** Andreas Brunauer, Andreas Koköfer, Otgon Bataar, Ilse Gradwohl-Matis, Daniel Dankl, Martin W Dünser

**Affiliations:** Department of Anesthesiology, Perioperative Care and Intensive Care Medicine, University Hospital Salzburg and Paracelsus Private Medical University, Müllner Hauptstrasse 48, 5020 Salzburg, Austria; Department of Emergency and Critical Care Medicine, Central State University Hospital, Marx Street, Ulaanbaatar, Mongolia

## Abstract

**Introduction:**

Liberal and overaggressive use of vasopressors during the initial period of shock resuscitation may compromise organ perfusion and worsen outcome. When transiently applying the concept of permissive hypotension, it would be helpful to know at which arterial blood pressure terminal cardiovascular collapse occurs.

**Methods:**

In this retrospective cohort study, we aimed to identify the arterial blood pressure associated with terminal cardiovascular collapse in 140 patients who died in the intensive care unit while being invasively monitored. Demographic data, co-morbid conditions and clinical data at admission and during the 24 hours before and at the time of terminal cardiovascular collapse were collected. The systolic, mean and diastolic arterial blood pressures immediately before terminal cardiovascular collapse were documented. Terminal cardiovascular collapse was defined as an abrupt (<5 minutes) and exponential decrease in heart rate (>50% compared to preceding values) followed by cardiac arrest.

**Results:**

The mean ± standard deviation (SD) values of the systolic, mean and diastolic arterial blood pressures associated with terminal cardiovascular collapse were 47 ± 12 mmHg, 35 ± 11 mmHg and 29 ± 9 mmHg, respectively. Patients with congestive heart failure (39 ± 13 mmHg versus 34 ± 10 mmHg; *P* = 0.04), left main stem stenosis (39 ± 11 mmHg versus 34 ± 11 mmHg; *P* = 0.03) or acute right heart failure (39 ± 13 mmHg versus 34 ± 10 mmHg; *P* = 0.03) had higher arterial blood pressures than patients without these risk factors. Patients with severe valvular aortic stenosis had the highest arterial blood pressures associated with terminal cardiovascular collapse (systolic, 60 ± 20 mmHg; mean, 46 ± 12 mmHg; diastolic, 36 ± 10 mmHg), but this difference was not significant. Patients with sepsis and patients exposed to sedatives or opioids during the terminal phase exhibited lower arterial blood pressures than patients without sepsis or administration of such drugs.

**Conclusions:**

The arterial blood pressure associated with terminal cardiovascular collapse in critically ill patients was very low and varied with individual co-morbid conditions (for example, congestive heart failure, left main stem stenosis, severe valvular aortic stenosis, acute right heart failure), drug exposure (for example, sedatives or opioids) and the type of acute illness (for example, sepsis).

**Electronic supplementary material:**

The online version of this article (doi:10.1186/s13054-014-0719-2) contains supplementary material, which is available to authorized users.

## Introduction

In patients with shock, it is imperative to reestablish adequate systemic blood flow and reverse tissue hypoperfusion [1]. This can typically be achieved by the administration of fluids and/or inotropes [1,2]. In severe arterial hypotension, however, the treatment response to these interventions may be too slow to rapidly increase arterial blood pressure levels to currently recommended levels (for example, a mean arterial blood pressure ≥65 mmHg [3,4]), despite reversal of the underlying pathology (for example, replenishment of hypovolemia, inotropic therapy). This is the reason why vasopressor drugs, such as norepinephrine, are commonly started liberally to avoid cardiovascular collapse due to ongoing arterial hypotension. Recent data, however, suggest that liberal use of vasoactive agents during the first hour may adversely affect mortality due to shock [5], particularly when systemic hypoperfusion is present, such as during hemorrhage [6]. Similarly, Subramanian *et al*. showed that restrictive vasopressor therapy during the first hours of shock can lead to improved tissue perfusion and less organ dysfunction, putatively by avoiding vasopressor-induced aggravation of systemic vasoconstriction and aggravation of tissue hypoperfusion [7]. Even marked delays in initiation of vasopressor therapy were associated with only a small increase in the risk of death in 8,640 patients with septic shock [8]. Because a strategy of restrictive vasopressor use during the early phase of shock implies that a certain degree of arterial hypotension is transiently tolerated to buy time so that definite hemorrhage control can be achieved (for example, in traumatic-hemorrhagic shock) [9] or ongoing fluid resuscitation and inotropic therapy can restore systemic blood flow (for example, in septic shock) [10]. When implementing such a concept of permissive hypotension with a primary focus on reversal of systemic hypoperfusion during early shock resuscitation, it would be helpful to know the range of arterial hypotension levels within which cardiovascular collapse occurs. Identification of these levels of arterial blood pressure, together with a certain safety margin, could help to guide emergent use of vasopressor drugs and avoid overaggressive use of vasoconstrictors in patients with systemic hypoperfusion [10,11].

In this retrospective cohort study, we aimed to identify the arterial blood pressure associated with terminal cardiovascular collapse in 140 patients who died in the intensive care unit (ICU) while being invasively monitored.

## Materials and methods

This study was designed as a retrospective cohort analysis. It included patients admitted to a 37-bed medical-surgical ICU of a tertiary university teaching hospital between July 2011 and April 2013. The study protocol was evaluated by the local ethics committee (Ethikkomission für das Bundesland Salzburg; 415-EP/73/203-2013), which waived the requirement for written informed consent because of the retrospective study design.

### Study patients and data collection

All critically ill patients who were invasively monitored and died in the ICU during the observation period were eligible for enrollment. The exclusion criteria were age younger than 18 years, pregnancy, organ donation following brain(stem) death, immediate death after withdrawal of ventricular assist device support and absence of continuous electrocardiographic or invasive arterial blood pressure measurements.

All study variables were extracted from the institutional electronic patient data management system (MetaVision; IMDSoft, Tel Aviv, Israel). This system prospectively collects demographic data and patient characteristics; hemodynamic and other vital parameters are collected at one minute intervals. The system uses median filtering at 1-minute intervals, which is an effective nonlinear, digital filtering process to eliminate artefacts due to a signal [12]. Data regarding drugs and fluids administered are entered manually into the database.

The following study variables were extracted from the database: age, sex, body mass index, premorbid conditions (known at ICU admission and/or diagnosed at autopsy), admission diagnosis, Simplified Acute Physiology Score (SAPS) II [13] and SAPS III [14] scores, end-of-life decisions, time from terminal cardiovascular collapse until death, cause of death, length of ICU stay and (wherever available) autopsy results. During the 24 hours before the terminal cardiovascular collapse, we documented the Sequential Organ Failure Assessment (SOFA) score [15], serum creatine kinase MB levels, as well as the presence and focus of sepsis [16], acute right heart failure (defined as low cardiac output due to increased pulmonary arterial pressure or decreased right ventricular contractility diagnosed by echocardiography or pulmonary artery catheter measurements) or shock (defined as need for catecholamine infusions to maintain tissue perfusion). At the time of the terminal cardiovascular collapse (see definition below), we collected the data for plethysmographic oxygen saturation; body temperature; heart rate; systolic, mean and diastolic arterial blood pressures (zeroing at the midchest level); central venous pressure; doses of catecholamines, vasopressin and/or levosimendan; exposure to sedative drugs and/or opioids; and last measurements of arterial lactate, base deficit, hemoglobin and partial arterial carbon dioxide tension.

### Determination of the arterial blood pressure associated with terminal cardiovascular collapse

**Figure 1:**
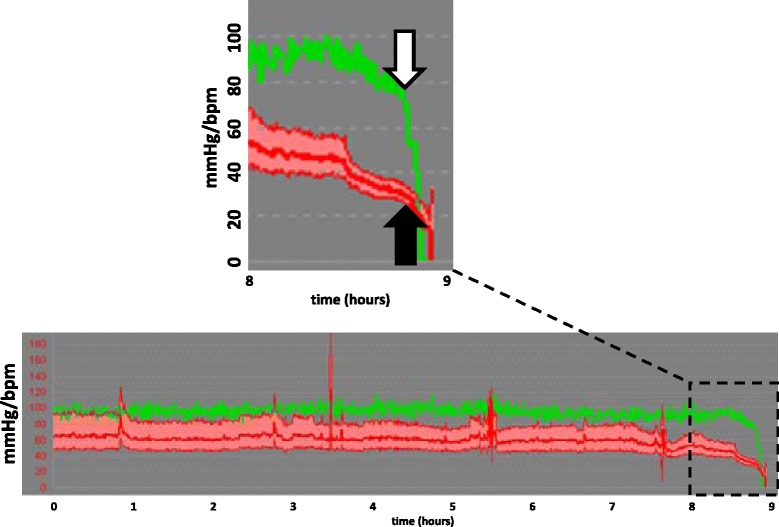
**Examples of the systolic, mean and diastolic arterial blood pressures at which terminal cardiovascular collapse occurred.** The arterial blood pressure (red curves) at which terminal cardiovascular collapse occurred is indicated by the black arrow), and abrupt and exponential decrease in heart rate (green curves) is indicated by the white arrow. bpm, Beats per minute.

The arterial blood pressure associated with terminal cardiovascular collapse was defined as the systolic, mean and diastolic arterial blood pressures immediately (within 1 minute) before terminal cardiovascular collapse occurred. Terminal cardiovascular collapse was defined as an abrupt (<5 minutes) and exponential decrease in heart rate (>50% compared to preceding values) followed by cardiac arrest (Figure 1). Patients in whom the drop in heart rate preceded the fall in arterial blood pressure were considered to have rhythmologic pathologies and were excluded from the analysis.

### Study endpoints

The primary endpoint of the study was to identify the systolic, mean and diastolic arterial blood pressure levels associated with terminal cardiovascular collapse in this study population. Secondary endpoints were to determine and compare these arterial blood pressure levels within and between the following subgroups: age >65 years, age >75 years, cardiac surgery, chronic arterial hypertension, congestive heart failure, coronary artery disease, diabetes mellitus, left main stem stenosis or equivalent, peripheral arterial occlusive disease, severe valvular aortic stenosis (grade 3 or 4), acute right heart failure, sepsis and shock. In addition, we analyzed whether the arterial blood pressure at terminal cardiovascular collapse was correlated with any of the following simultaneously measured variables: age, SOFA score, hemoglobin, partial arterial carbon dioxide tension, plethysmographic oxygen saturation, body temperature and exposition to sedative drugs and/or opioids.

### Statistical analysis

All statistical analyses were performed using the PASW Statistics 18 software package (SPSS, Chicago, IL, USA). Following plausibility testing of the dataset, the normality distribution was checked by using the Kolmogorov-Smirnov test and was approximately fulfilled by all variables. Descriptive methods were used to present patient characteristics and the arterial blood pressures associated with terminal cardiovascular collapse in the study population and subgroups. The unpaired Student’s *t*-test was used to compare these arterial blood pressure levels between subgroups. Bivariate correlations calculated by applying the Pearson correlation coefficient were used to evaluate the relationship between the arterial blood pressure associated with terminal cardiovascular collapse and predefined cofactors. *P*-values <0.05 were considered to indicate statistical significance. All parameters are presented as mean values ± SD, if not indicated otherwise.

## Results

Two hundred thirteen ICU patients died during the observation period. Of these, no one was aged <18 years or pregnant, but electrocardiographic or invasive blood pressure measurements were not recorded in 22 patients. Nine patients died after organ donation following brain(stem) death, and five died after withdrawal of venoarterial extracorporeal membrane oxygenation therapy. Of the remaining 177 patients, 27 had rhythmological pathologies in the terminal phase. In ten patients, the arterial blood pressure at terminal cardiovascular collapse could not be determined. Following exclusion of these patients, 140 patients were included in the final analysis (Tables 1 and 2). In 120 patients (85.7%), end-of-life decisions were made to withhold or withdraw invasive organ support. At the time of terminal cardiovascular collapse, 113 patients (80.7%) were receiving sedatives, opioids or both.

**Table 1:** **Characteristics of the study population**

**Characteristics**		**Patient data (** ***N*** **= 140)**
Age (yr)		72.8 ± 13.2
Male sex (*n* (%))		87 (62.1)
Body mass index (kg/m^2^)		26.1 ± 5.5
Comorbid conditions (*n* (%))		
	Chronic arterial hypertension	78 (55.7)
	Coronary artery disease	57 (40.7)
	Congestive heart failure	27 (19.3)
	Severe aortic stenosis	3 (2.1)
	Chronic renal failure	26 (18.6)
	Diabetes mellitus	26 (18.6)
	Peripheral arterial occlusive disease	15 (10.7)
Admission diagnosis (*n* (%))		
	Post–cardiac arrest	26 (18.6)
	Shock of any origin	22 (15.7)
	Abdominal disease	21 (15)
	Respiratory insufficiency	20 (14.3)
	Trauma	12 (8.6)
	Post–cardiac surgery	10 (7.1)
	Gastrointestinal hemorrhage	7 (5)
	Miscellaneous	22 (15.6)
SAPS II^a^ (points)		53 ± 20
SAPS III^a^ (points)		73 ± 18
Intensive care unit length of stay (days)		7.1 ± 9.6
Autopsy performed (*n* (%))		84 (60)

^a^SAPS, Simplified Acute Physiology Score. Data are presented as mean values ± SD, if not otherwise indicated.

**Table 2:** **Clinical data 24 hours before and at the time of terminal cardiovascular collapse, as well as causes of death**

		**Clinical data**
**24 hours before terminal cardiovascular collapse**		
Sepsis (*n* (%))		34 (24.3)
Sepsis focus (*n* (%))		
	Abdomen	9 (6.4)
	Soft tissue/joints	7 (5)
	Lungs	3 (2.1)
	Primary bacteremia	2 (1.4)
	Miscellaneous	18 (9.3)
Shock (*n* (%))		87 (62.1)
Acute right heart failure (*n* (%))		22 (15.7)
Sequential Organ Failure Assessment score (points)		12 ± 4
**At the time of terminal cardiovascular collapse**		
Base deficit (mmol/L)		−5.1 ± 8
Arterial lactate (mmol/L)		5.4 ± 4.8
Hemoglobin (mg/dl)		9.6 ± 1.9
Partial arterial carbon dioxide tension (mmHg)		52 ± 30
Plethysmographic oxygen saturation (%)		77 ± 16
Body temperature (°C)		36.9 ± 1.8
Heart rate (beats/min)		88 ± 24
Central venous pressure (mmHg)		14 ± 7
Catecholamine infusion (*n* (%))		84 (60)
Vasopressin infusion (*n* (%))		6 (4.3)
Levosimendan infusion (*n* (%))		5 (3.6)
**Cause of death**		
Cardiovascular failure (*n* (%))		84 (60)
Multiple organ dysfunction (*n* (%))		29 (20.7)
Respiratory failure (*n* (%))		15 (10.7)
Severe ischemic encephalopathy (*n* (%))		12 (8.6)

Data are presented as mean values ± SD, if not otherwise indicated.

**Figure 2:**
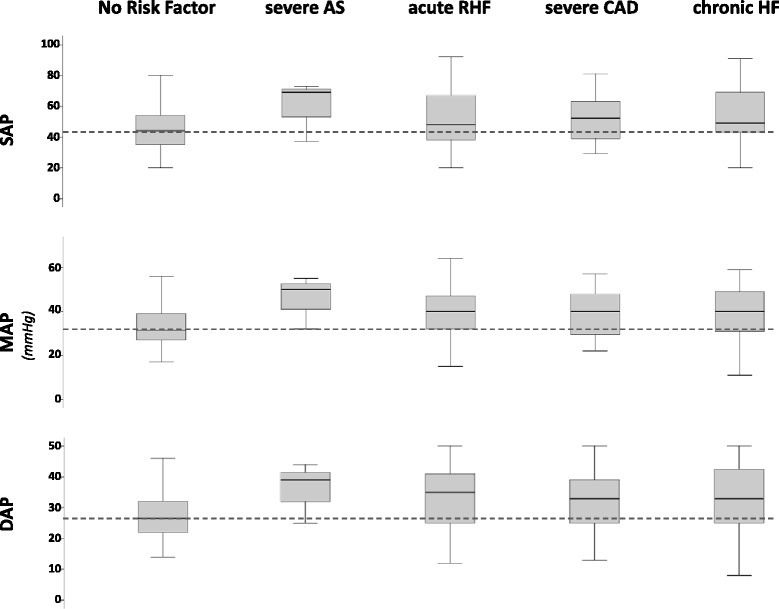
**Box plots showing arterial blood pressures associated with terminal cardiovascular collapse in patients with and without specific risk factors.** AS, Arterial stenosis; CAD, Coronary artery disease; DAP, Diastolic arterial pressure; HF, Heart failure; MAP, Mean arterial pressure; RHF, Right heart failure; SAP, Systolic arterial pressure. Boxed areas indicate median values with interquartile ranges. Error bars indicate minimum and maximum values. The dashed lines represents median values in patients with no risk factor.

The intraobserver and interobserver agreement for identification of the arterial blood pressure associated with terminal cardiovascular collapse were 94% and 88%, respectively. The systolic, mean and diastolic arterial blood pressures (mean ± SD) associated with terminal cardiovascular collapse were 47 ± 12 mmHg, 35 ± 11 mmHg and 29 ± 9 mmHg, respectively (Table 3). The time from cardiovascular collapse to death was 31 ± 30 minutes. Patients with congestive heart failure, left main stem stenosis or acute right heart failure had higher mean arterial blood pressures at terminal cardiovascular collapse than patients without these risk factors. Among the patients with congestive heart failure, 77.8% had coronary artery disease and 44.4% had left main stem stenosis. Patients with severe valvular aortic stenosis had the highest arterial blood pressure at terminal cardiovascular collapse, but this difference missed the significance level (Table 3, Figure 2). Patients with sepsis during the 24 hours before terminal cardiovascular collapse exhibited lower arterial blood pressure at terminal cardiovascular collapse than patients without sepsis. The incidences of comorbid conditions (congestive heart failure, 8.8% versus 22.6%, *P* = 0.09; left main stem stenosis, 8.8% versus 18.9%, *P* = 0.2; severe valvular aortic stenosis, 2.9% versus 1.9%, *P* = 0.57) and acute right heart failure (17.6% versus 15.1%, *P* = 0.79), as well as exposition to sedatives and/or opioids during the terminal phase (88.2% versus 78.3%, *P* = 0.32), did not differ between patients with sepsis and those without sepsis. No differences in arterial blood pressure at terminal cardiovascular collapse between the remaining subgroups were observed (Table 3).

**Table 3:** **Arterial blood pressures at terminal cardiovascular collapse**

	**Systolic arterial blood pressure**			**Mean arterial blood pressure**			**Diastolic arterial blood pressure**		
	**(Total population, 47 ± 17)**			**(Total population, 35 ± 11)**			**(Total population, 29 ± 9)**		
**Pre-defined risk factors**	**Risk factor**	**No risk factor**	***P*** **-value**	**Risk factor**	**No risk factor**	***P*** **-value**	**Risk factor**	**No risk factor**	***P*** **-value**
Age >65 yr (*n* = 103)	47 ± 17	47 ± 18	0.99	35 ± 11	34 ± 12	0.64	29 ± 9	28 ± 10	0.61
Age >75 yr (*n* = 66)	50 ± 17	45 ± 17	0.06	37 ± 10	33 ± 11	0.05	30 ± 9	28 ± 10	0.16
Cardiac surgery (*n* = 10)	51 ± 19	47 ± 17	0.53	35 ± 12	35 ± 11	0.95	27 ± 11	29 ± 9	0.69
Chronic arterial hypertension (*n* = 78)	49 ± 17	45 ± 17	0.12	36 ± 11	33 ± 10	0.18	29 ± 10	28 ± 9	0.29
Congestive heart failure (*n* = 27)	53 ± 20	46 ± 16	0.06	39 ± 13	34 ± 10	0.04^a^	31 ± 12	28 ± 8	0.17
Coronary artery disease (*n* = 57)	48 ± 18	47 ± 17	0.76	36 ± 12	34 ± 10	0.34	30 ± 10	28 ± 8	0.27
Diabetes mellitus (*n* = 26)	47 ± 17	48 ± 17	0.81	35 ± 11	35 ± 11	0.98	29 ± 9	28 ± 9	0.71
Left main stem stenosis (*n* = 23)	53 ± 16	46 ± 17	0.08	39 ± 11	34 ± 11	0.03^a^	32 ± 10	28 ± 9	0.07
PAOD^b^ (*n* = 15)	43 ± 17	48 ± 17	0.34	33 ± 10	35 ± 11	0.63	27 ± 8	29 ± 9	0.62
Acute right heart failure (*n* = 22)	53 ± 20	46 ± 16	0.1	39 ± 13	34 ± 10	0.03^a^	32 ± 11	28 ± 9	0.06
Sepsis (*n* = 34)	42 ± 12	49 ± 18	0.005^a^	30 ± 8	36 ± 11	0.001^a^	25 ± 8	30 ± 9	0.02^a^
Severe aortic stenosis (*n* = 3)	60 ± 20	47 ± 17	0.2	46 ± 12	34 ± 11	0.08	36 ± 10	28 ± 9	0.16
Shock (*n* = 87)	48 ± 17	47 ± 17	0.73	35 ± 11	34 ± 10	0.36	29 ± 10	27 ± 8	0.14

^a^Significant difference. ^b^PAOD, Peripheral arterial occlusive disease. All units are millimeters of mercury. Data are presented as mean values ± SD, if not indicated otherwise.

Patients exposed to sedatives and/or opioids at the time of terminal cardiovascular collapse had lower systolic (46 ± 17 mmHg versus 53 ± 15 mmHg; *P* = 0.048) and mean arterial blood pressures (34 ± 11 mmHg versus 39 ± 10 mmHg; *P* = 0.03) than patients without sedative and/or opioid administration. The correlation analysis revealed a significant indirect relationship (Table 4). No significant correlation was observed between the arterial blood pressure at terminal cardiovascular collapse and other cofactors investigated (Table 4). There were also no relationships between the serum creatine kinase MB and arterial lactate levels during the last 24 hours with the mean arterial blood pressure at laboratory measurement or the arterial blood pressure at terminal cardiovascular collapse (Additional file 1: Figure S1).

**Table 4:** **Bivariate correlations between arterial blood pressures at terminal cardiovascular collapse and predefined cofactors** ^**a**^

**Cofactor**	**Systolic arterial blood pressure**		**Mean arterial blood pressure**		**Diastolic arterial blood pressure**	
	**Pearson coefficient**	***P*** **-value**	**Pearson coefficient**	***P*** **-value**	**Pearson coefficient**	***P*** **-value**
Age (yr)	0.1	0.24	0.11	0.2	0.07	0.38
SpO_2_ (%)	−0.06	0.54	0.02	0.82	0.05	0.63
Temperature (°C)	−0.08	0.41	−0.09	0.31	−0.13	0.17
Hemoglobin (g/dl)	0.03	0.69	0.04	0.69	<0.01	0.99
PaCO_2_ (mmHg)	0.15	0.08	0.08	0.38	0.03	0.7
SOFA score (points)	−0.04	0.61	0.06	0.48	0.14	0.11
Sedative and/or opioid exposure	−0.17	0.048^b^	−0.185	0.03^b^	−0.16	0.06

^a^PaCO_2_, partial carbon dioxide pressure; SOFA, Sequential Organ Failure Assessment; SpO_2_, plethysmographic oxygen saturation. ^b^Significant correlation.

## Discussion

In this retrospective cohort study including 140 critically ill patients, the mean systolic, mean and diastolic arterial blood pressures associated with terminal cardiovascular collapse were 47, 35 and 29 mmHg, respectively. Patients with congestive heart failure, left main stem stenosis, acute right heart failure or severe valvular aortic stenosis exhibited higher arterial blood pressures at terminal cardiovascular collapse than patients without these risk factors. Patients with sepsis had lower arterial blood pressures than patients without sepsis. Similarly, systolic and mean arterial blood pressures were lower in patients exposed to sedatives and/or opioids at the time of terminal cardiovascular collapse.

In our analysis, we identified the arterial blood pressure associated with terminal cardiovascular collapse as indicated by an abrupt and exponential drop in heart rate. It is conceivable that, at these low arterial blood pressure levels, coronary perfusion was reduced to such an extent that global myocardial hypoperfusion and ischemia occurred. As we did not measure coronary blood flow in our population, no association between the arterial blood pressure at terminal cardiovascular collapse and the lower threshold of coronary autoregulation can be made. From a physiologic perspective, it is likely that terminal cardiovascular collapse occurs at arterial blood pressures well below the lower coronary autoregulation limit and that adverse events such as coronary or end-organ hypoperfusion occur at higher arterial blood pressures than the ones identified in this study [17]. Therefore, the arterial blood pressures associated with terminal cardiovascular collapse should by no means implicate safe values or be regarded as a resuscitation endpoint. They should instead be considered as the lowest end of cardiovascular regulation in a mixed critically ill patient population. Even when transiently applying the strategy of permissive hypotension, these arterial blood pressure levels should never be reached or tolerated, but rather an individual safety limit (for example, one or two standard deviations) above these arterial blood pressure levels should be maintained.

Five subgroups presented with arterial blood pressures at terminal cardiovascular collapse that were different from those in the remaining population. The respective mean arterial blood pressure levels differed by approximately 5 mmHg between patients with and those without acute right heart failure. This finding is in line with clinical observations that patients with acute right heart failure require an adequate perfusion pressure to maintain right ventricular function [18,19]. Patients with congestive heart failure and left main stem stenosis exhibited mean arterial blood pressures at terminal cardiovascular collapse that were, on average, 3 to 7 mmHg above those of patients without these risk factors. As three-fourths of patients with congestive heart failure had coronary artery disease and one-half of them from left main stem stenosis or an equivalent, the two subgroups are likely to share similar reasons for higher arterial pressure requirements. Higher arterial blood pressures are similarly required to maintain coronary blood flow in patients with significant coronary stenosis [20,21]. Although patients with severe valvular aortic stenosis exhibited the highest arterial blood pressures at terminal cardiovascular collapse in this cohort, comparisons with the remaining population failed to reach the significance level, owing to the small number of patients (*n* = 3). Also, in severe aortic stenosis, arterial blood pressure is a key determinant of coronary perfusion [22,23], which explains why these patients required arterial blood pressures 8 to 13 mmHg higher than patients without severe aortic stenosis. Patients with sepsis experienced terminal cardiovascular collapse only when arterial blood pressures were 5 to 7 mmHg lower than in the remaining population. This observation is striking and cannot be explained by differences in comorbid conditions or the incidence of acute right heart failure in these patients. It may be hypothesized that one reason for this observation is that sepsis patients had higher cardiac output levels than patients without sepsis and could thus tolerate lower arterial blood pressures. However, our study cannot prove this hypothesis, because cardiac output was not measured in any study patient during the terminal phase. Although no further differences in arterial blood pressures at terminal cardiovascular collapse could be identified, our analysis may have included too few patients to uncover minor differences between other subgroups and some of the observed variations between subgroups may have equally resulted from small patient numbers in each group.

Another interesting finding of this analysis is that patients receiving sedatives and/or opioids during the terminal phase experienced terminal cardiovascular collapse at significantly lower arterial blood pressures than patients who were not exposed to such drugs. This observation may have important practical implications because terminal cardiovascular collapse in awake patients or patients not exposed to sedative agents or opioids may occur at relevantly higher arterial blood pressure levels (5 to 8 mmHg on average) than in sedated patients. Other investigators have postulated that sedative agents and opioids can reduce oxygen requirements and hence increase the body’s tolerance to hypoxia and arterial hypotension [24], as may have been the case in our cohort. These results are in accordance with data showing that a 1 mg/hr increase of the benzodiazepine dose after withdrawal of life support increased the time until death by 13 minutes [25]. Further correlation analyses between arterial blood pressure at terminal cardiovascular collapse and various other cofactors did not reveal significant relationships. Several reasons may account for this unexpected finding. First, 140 patients may still have been too small a study population to detect some correlations. Second, cofactors that were not included in our correlation analysis may play a relevant role. For example, it is likely that the individual hemodynamic management and duration of preceding arterial hypotension, particularly the time spent with arterial blood pressures below the lower coronary autoregulation threshold, may affect the arterial blood pressure at terminal cardiovascular collapse.

When interpreting the results of this study, additional limitations need to be noted. This was a retrospective analysis and may thus contain methodological weaknesses such as missing values and heterogeneous datasets. A relevant selection bias may have been introduced into the analysis in that 20.9% of patients who originally met the inclusion criteria were excluded because their arterial blood pressure at terminal cardiovascular collapse could not be determined or because their terminal cardiovascular collapse was caused by rhythmologic pathologies. Although we failed to identify an arterial blood pressure at which rhythmologic pathologies occurred in patients excluded from this analysis (data not shown), it is possible that such terminal rhythm disturbances might have been the consequence of coronary hypoperfusion and that a delicate group of patients exclusively sensitive to low arterial blood pressure levels was excluded from our analysis.

## Conclusions

The arterial blood pressure associated with terminal cardiovascular collapse in critically ill patients was very low and varied with individual comorbid conditions (for example, congestive heart failure, left main stem stenosis, severe valvular aortic stenosis, acute right heart failure), drug exposure (for example, sedatives and/or opioids) and the type of acute illness (for example, sepsis).

## Key messages

- The arterial blood pressure associated with terminal cardiovascular collapse in critically ill patients was very low at 47 ± 12 mmHg systolic, 35 ± 11 mmHg mean and 29 ± 9 mmHg diastolic arterial blood pressure.
- Relevant exceptions were patients with congestive heart failure, left main stem stenosis, severe valvular aortic stenosis or acute right heart failure.
- Sepsis patients and patients receiving sedatives and/or opioids exhibited even lower arterial blood pressures at terminal cardiovascular collapse.

## Additional file

Additional file 1: Figure S1. Arterial lactate levels and critical MAP values in patients stratified into different MAP levels at last lactate measurement (242 ± 280 minutes before cardiovascular collapse).

## Abbreviations

AS: Arterial stenosis
CAD: Coronary artery disease
DAP: Diastolic arterial pressure
HF: Heart failure
ICU: Intensive care unit
MAP: Mean arterial pressure
PaCO_2_: Partial carbon dioxide pressure
PAOD: peripheral arterial occlusive disease
RHF: Right heart failure
SpO_2_: Plethysmographic oxygen saturation
SAP: Systolic arterial pressure
SAPS: Simplified Acute Physiology Score
SOFA: Sequential Organ Failure Assessment
