# Supplementary figures and images for: The arterial blood pressure associated with terminal cardiovascular collapse in critically ill patients: a retrospective cohort study
Source: Crit Care. 2014 Dec 19;18(6):719. doi: 10.1186/s13054-014-0719-2 (PMC4299308; doi:10.1186/s13054-014-0719-2)

## Slide 1
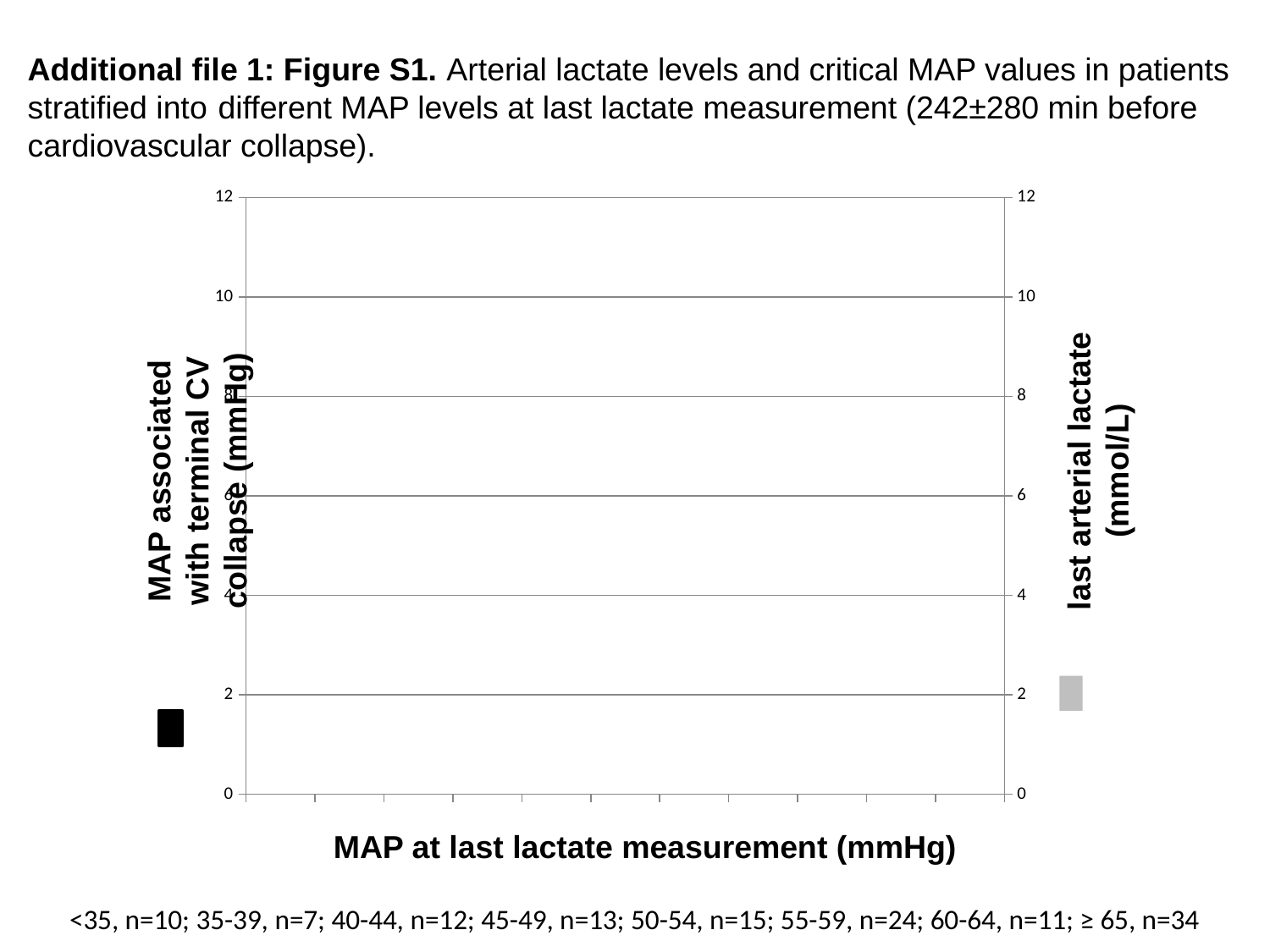

## Slide 2
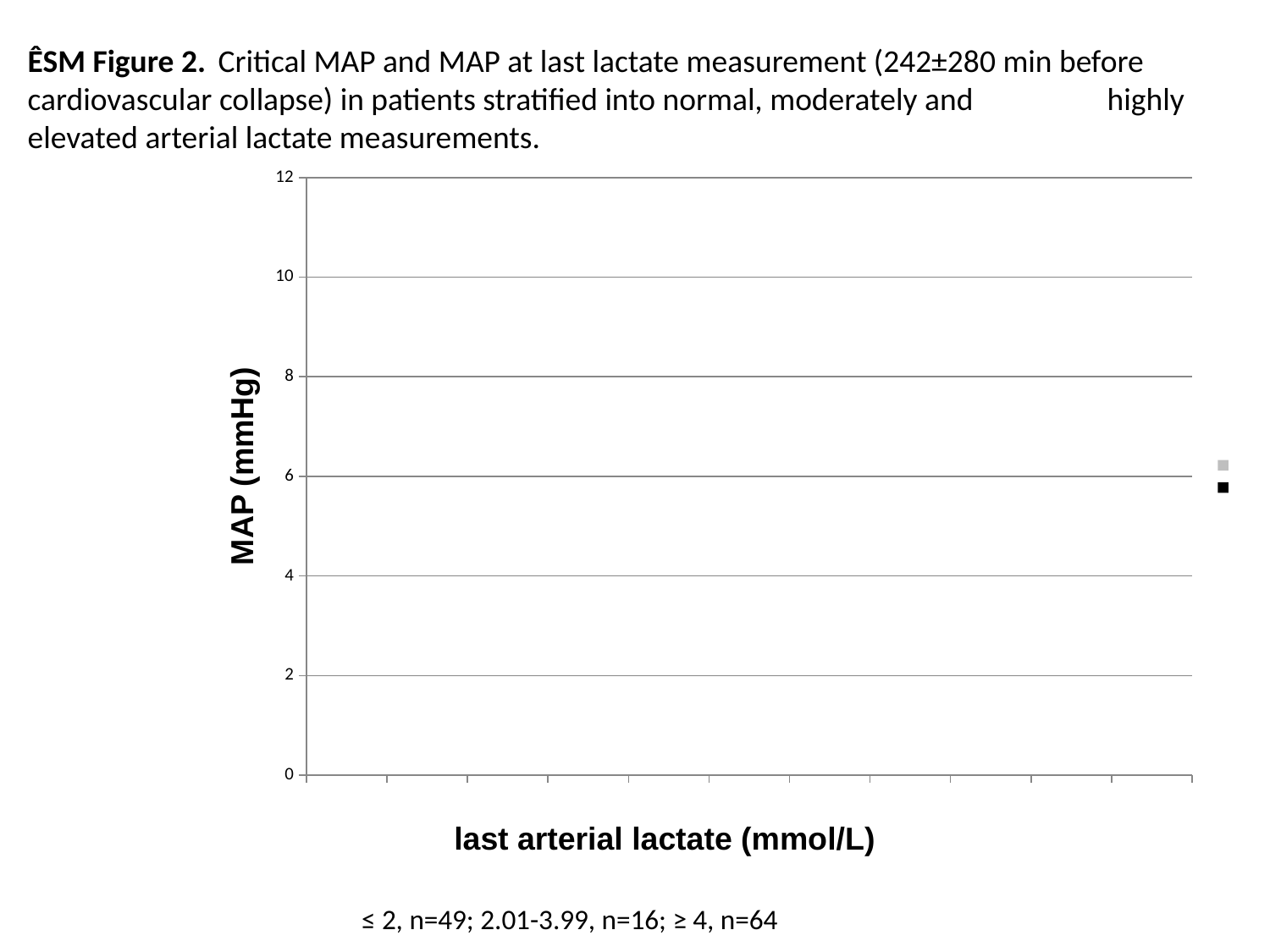

## Slide 3
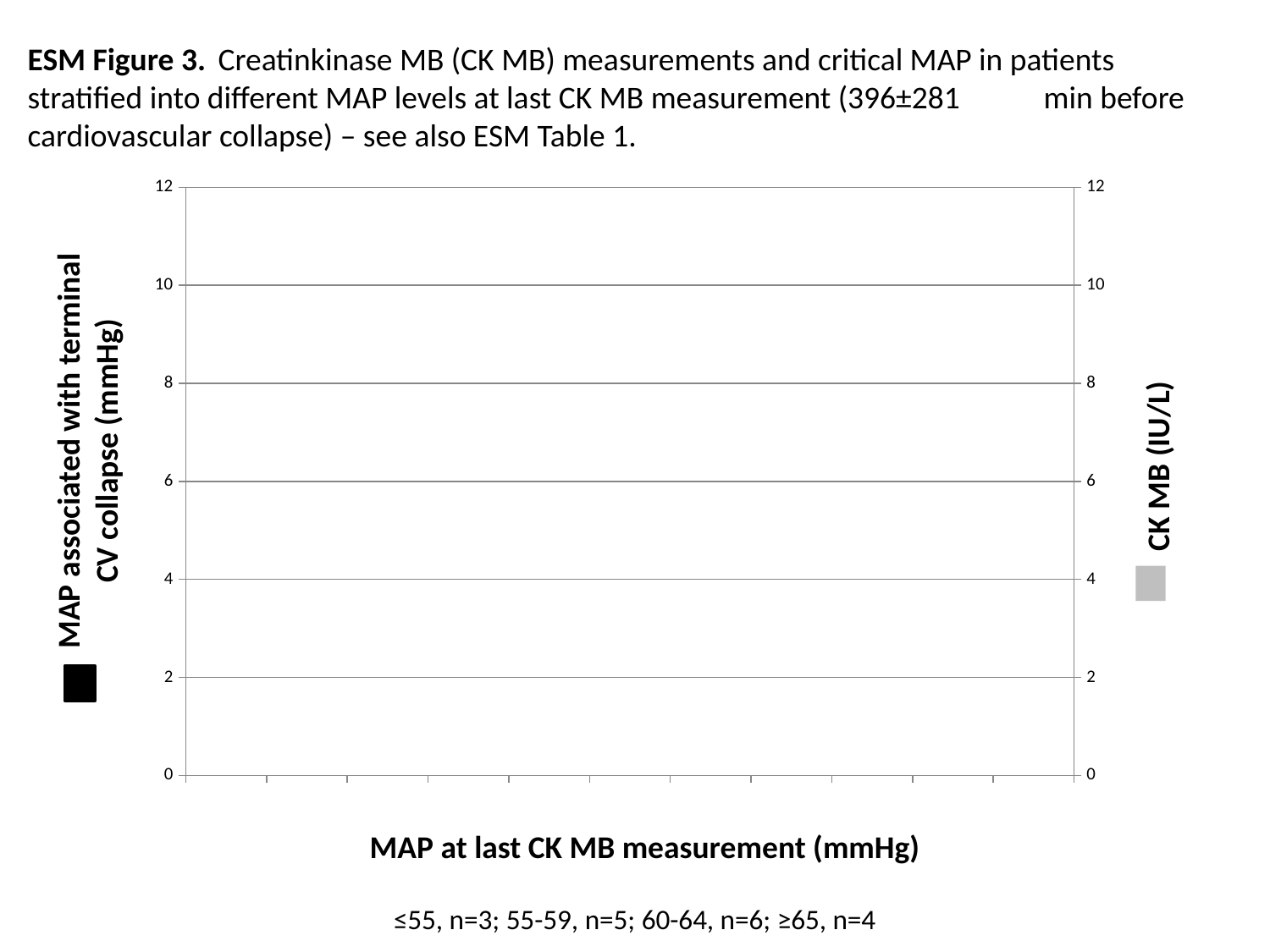

## Slide 4
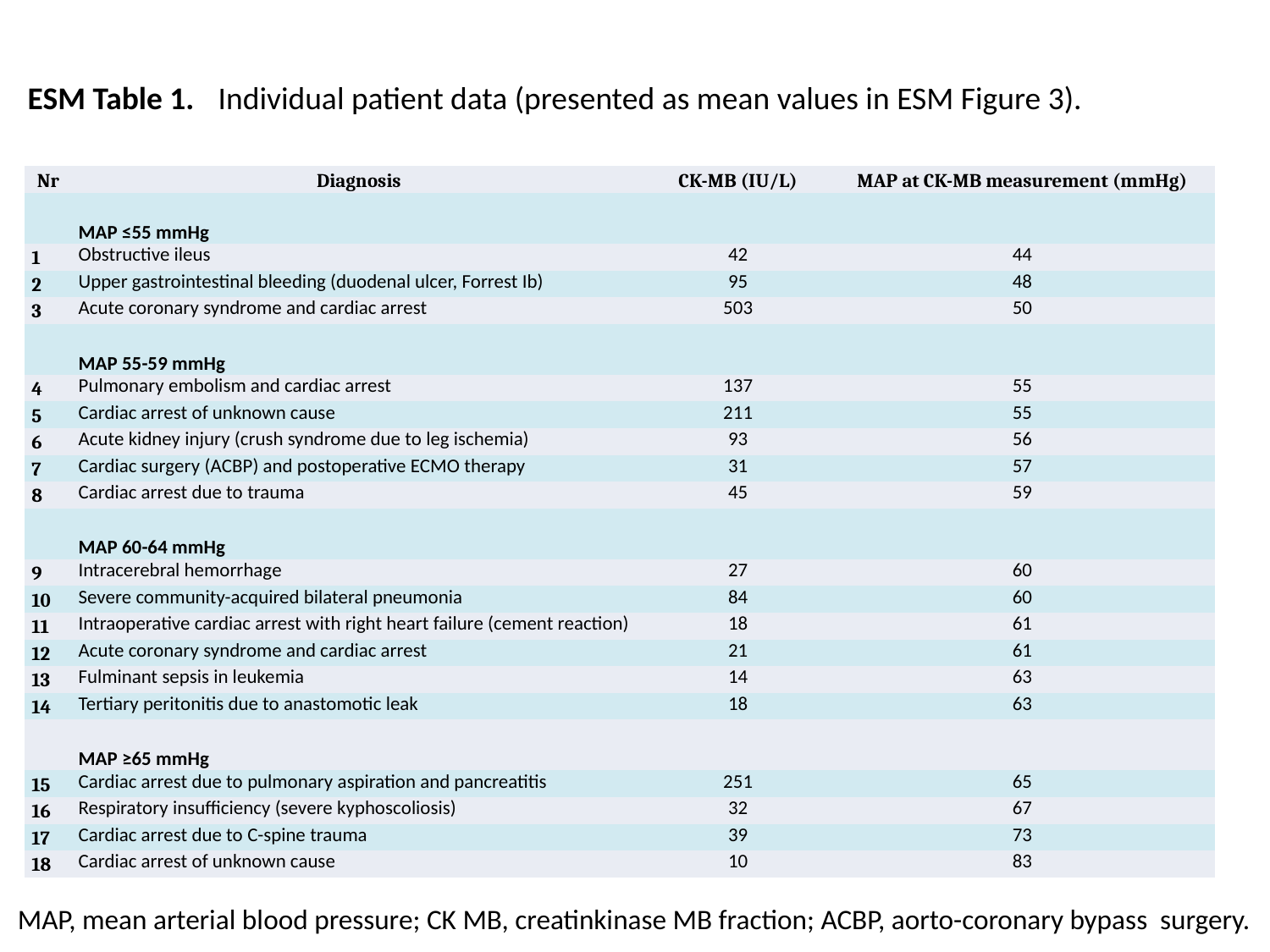

## Slide 5
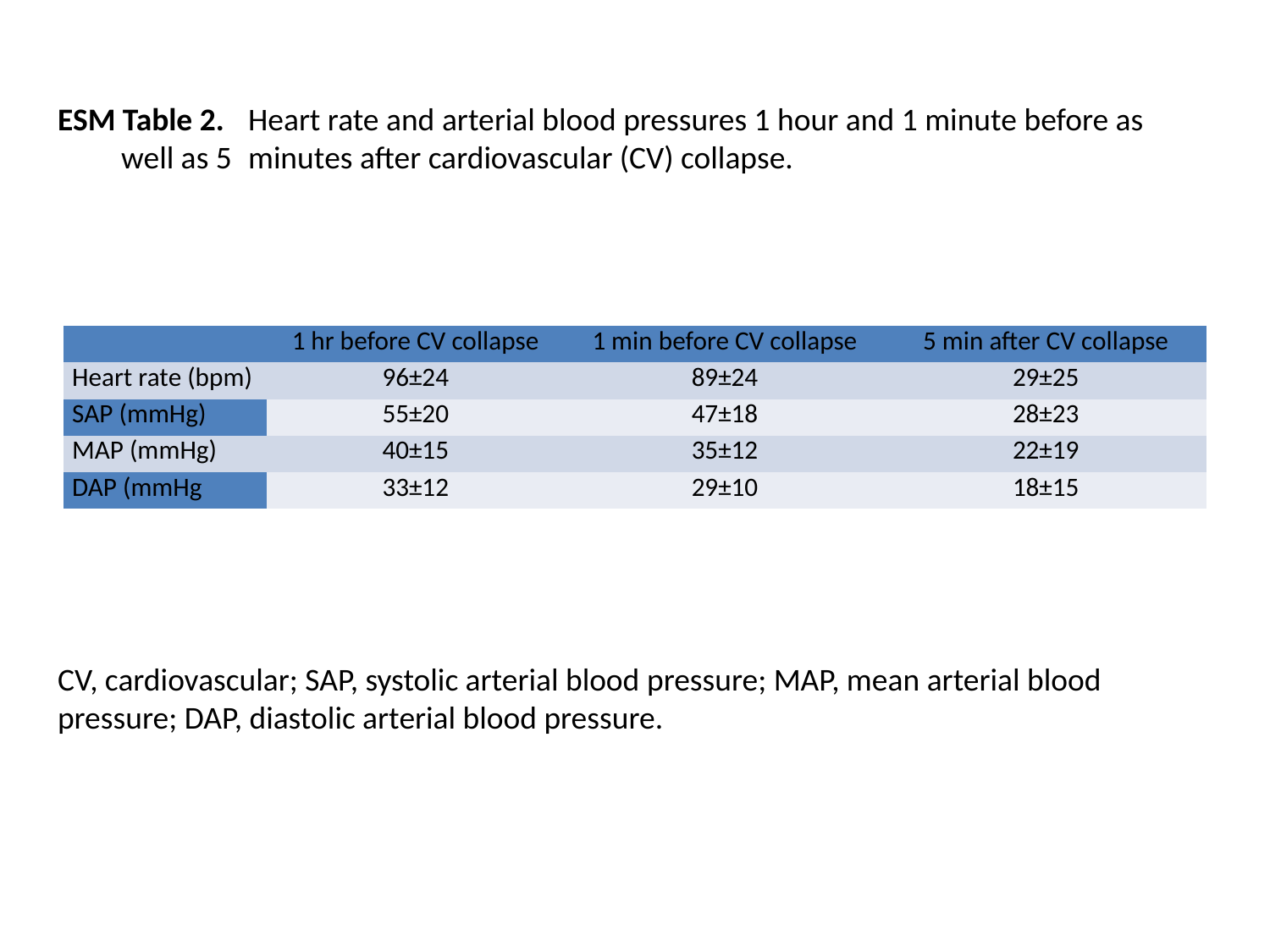

Supplement: Additional file 1: Figure S1. — Arterial lactate levels and critical MAP values in patients stratified into different MAP levels at last lactate measurement (242 ± 280 minutes before cardiovascular collapse). [file 13054_2014_719_MOESM1_ESM.pptx]
